# On the state of crystallography at the dawn of the electron microscopy revolution

**DOI:** 10.1016/j.sbi.2017.06.005

**Published:** 2017-10

**Authors:** Matthew K .Higgins, Susan M Lea

**Affiliations:** 1 Department of Biochemistry, University of Oxford, South Parks Road, Oxford OX1 3QU, UK; 2 Sir William Dunn School of Pathology, University of Oxford, South Parks Road, Oxford OX1 3RE, UK

## Abstract

While protein crystallography has, for many years, been the most used method for structural analysis of macromolecular complexes, remarkable recent advances in high-resolution electron cryo-microscopy led to suggestions that ‘the revolution will not be crystallised’. Here we highlight the current success rate, speed and ease of modern crystallographic structure determination and some recent triumphs of both ‘classical’ crystallography and the use of X-ray free electron lasers. We also outline fundamental differences between structure determination using X-ray crystallography and electron microscopy. We suggest that crystallography will continue to co-exist with electron microscopy as part of an integrated array of methods, allowing structural biologists to focus on fundamental biological questions rather than being constrained by the methods available.

## Highlights

• Protein crystallography plays a major role in integrative structural biology.
• XFEL methods bring new opportunities for structural analysis of dynamic systems.
• Crystallography and electron microscopy will be complementary into the future.

**Current Opinion in Structural Biology** 2017, **46**:95–101

This review comes from a themed issue on **Biophysical methods: behind the scenes of the cryo-EM revolution**

Edited by **Carol Robinson** and **Carla Schmidt**

For a complete overview see the Issue and the Editorial

Available online 4th July 2017

**http://dx.doi.org/10.1016/j.sbi.2017.06.005**

0959-440X/© 2017 The Authors. Published by Elsevier Ltd. This is an open access article under the CC BY license (http://creativecommons.org/licenses/by/4.0/).

## Introduction

Since the 1950s, the method of choice for the determination of protein structures has been X-ray crystallography, and innovations in sample handling, X-ray sources, detectors and software have since dramatically reduced the time taken to determine a structure [1]. Data collection at a ‘standard’ synchrotron source generally takes only a few seconds [2], while automated pipelines facilitate data collection [3], and allow many structures to be solved without intervention by the user [3, 4]. The high level of automation and speed of the experiment have revolutionized how crystallography is performed, making it standard to collect data from several tens to hundreds of crystals and allowing determination of structures from crystal systems that would previously have been considered intractable. Recent advances include quick data collection, free at the point of access synchrotron facilities and simple to use or highly automated beamlines [5] and software [3]. These have contributed to an ever growing number of coordinate sets deposited in the Protein Data Bank. Indeed crystallography is still by far the most used method for structure determination (Table 1).

**Table 1:** Structures deposited in the protein data bank (www.rcsb.org) determined by the major structural methods

Method	2015	2016
Solution NMR	346	450
Solid state NMR	11	7
X-ray	7662	9964
XFEL	15	57
EM	216	410

Advances in both synchrotron hardware and in software suites have made the determination of novel structures more streamlined, with a massive case history helping the community to employ the best strategies to collect data [6]. While experimental phasing previously relied on introduction of non-native heavy atoms into the macromolecule under study, long wavelength beams are allowing phasing using weak anomalous signal from naturally occurring atoms, such as sulphur, making resolution of ‘the phase problem’ increasingly routine [7]. Coupling these weak signals with molecular replacement, using search models derived from the latest protein modeling tools, is providing increased power for *de novo* structure determination [8]. Advances in automatic data collection are also improving the throughput of crystallography as a tool for drug design. For systems that generate well diffracting crystals, screening platforms, including semi-automated crystal mounting, together with high-throughput automatic data collection and processing, allow rapid screening of small molecules and molecular fragments, to identify those with promise as part of molecules of medicinal value (for example http://www.diamond.ac.uk/Beamlines/Mx/Fragment-Screening.html) [9]. It is therefore easier to both determine a novel structure and to exploit this structure for therapeutic use.

While the ability to grow a crystal remains limiting for standard crystallography, what defines a useful crystal is in constant flux, with the absolute size of crystals, and their required degree of order, continuously decreasing. Improvements to synchrotron facilities include the availability of microfocus sources, such as beamline I24 at Diamond Light Source [10], providing small and intense beams to coax diffraction from crystals a few micrometers across. These crystals can even be imaged in crystallization plates or collected onto mesh supports, with small numbers of images collected from individual crystals, and complete diffraction patterns obtained by piecing these together [11, 12, 13]. Serial crystallography, with single diffraction patterns collected from microcrystals or nanocrystals, and data collection using X-ray free-electron lasers, are turning the size restrictions on crystals on their head, making small crystals desirable, and allowing collection of diffraction data from crystals at room temperature, untainted by beam-induced radiation damage. These methods have great power to determine structural changes induced in a macromolecule by light or by ligand.

In this review we will briefly highlight how these developments place X-ray techniques at the heart of integrated structural biology and will describe how fundamental differences in the basis of structure determination by different methods mean that all the structural techniques will continue to have roles to play for the foreseeable future. While there is no doubt that advances in electron microscopy are opening exciting new possibilities for the structural biologist, claims of the demise of crystallography seem premature, if not unfounded.

## Crystallography at the heart of integrative structural biology: some recent triumphs

**Figure 1:**
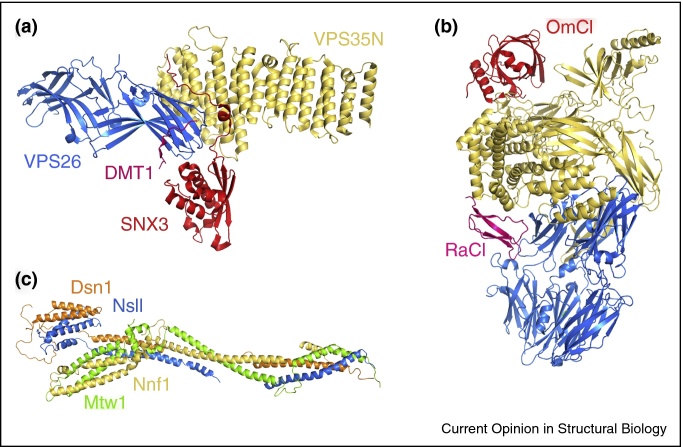
Crystallography addressing major problems in cell biology. **(a)** The structure of the retromer complex gives insight into cargo recruitment (PDB code: 5F0P) [14]. **(b)** Crystal structure of human Complement C5 with two inhibitors derived from tick saliva, *Ornithodoros moubata* OmCI and *Dermacentor andersoni* RaCI3 (PDB code: 5HCC). Adapted from [15]. **(c)** The structure of the MIND complex and the assembly of yeast kinetochores (PDB code: 5T58) [16].

Many exciting studies over recent years illustrate the continuing power of classical crystallography to underpin integrative structural and cellular science. Examples that have caught the eyes of the authors include structural analysis of cellular trafficking [14], complement regulation [15], kinetochore assembly [16] and nuclear pore formation [17] (Figure 1). Crystal structures of a large complex from the retromer system involved in membrane protein recycling, supported by small angle X-ray scattering, and biophysical and cellular analysis, have revealed new insight into the process by which signal recognition leads to membrane recruitment in this trafficking system [14]. Novel crystal structures, combined with NMR, electron microscopy and functional and biophysical analysis have shown how antibodies and proteins from tick salivary glands can inhibit critical complement pathways [15]. Structures of the MIND complex, determined using powerful crystallographic tools to overcome the challenges associated with anisotropic data and small crystals, have given important insights into kinetochore assembly [16]. Finally, a study has generated a molecular model for the mRNA export platform of the nuclear pore complex using a combination of mass spectrometry, cross-linking, electron microscopy and molecular modeling, allowing the assembly of previously determined crystal structures into a larger assembly [17]. Each of these studies highlights how modern synchrotrons, advanced detectors and the latest generation of processing software are allowing determination of increasingly complex structures and show how crystallography can be integrated with other structural and cellular methods to answer important biological questions.

**Figure 2:**
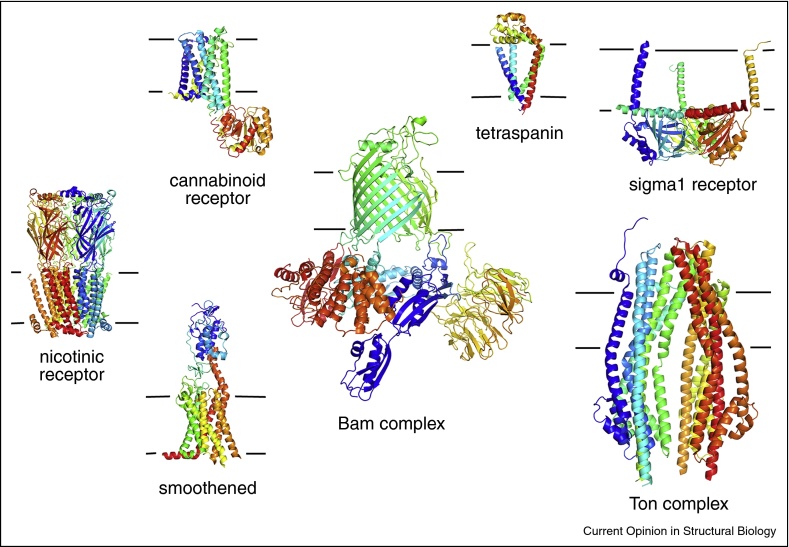
A cornucopia of membrane protein crystal structures from 2016. The structures of the cannabinoid receptor (PDB code: 5U09); tetraspanin (PDB code: 5TCX); the sigma1 receptor (PDB code: 5HK1), the nictonic receptor (PDB code: 5KXI); smoothened (PDB code: 5L7D); the Bam complex (PDB code: 5D0Q) and the Ton complex (PDB code: 5SV0). Lines represent the approximate position of the membrane.

The world of membrane protein crystallography also continues to advance. Here, developments in crystallization of proteins embedded in lipidic cubic phase, mimicking the native membrane environment, have been widely adopted. Used in conjunction with microfocus and long wavelength beams to allow collection of data from these frequently tiny crystals, this has provided unparalleled insight into proteins that function within the membrane environment. 2016 alone saw publication of structures of the cannabinoid receptor [18, 19], the human α4β2 nicotinic receptor [20], the developmental signal transducer smoothened [21], large bacterial membrane protein complexes Ton [22] and Bam [23, 24] and human tetraspanin [25] and sigma receptor [26] (Figure 2). Two of these studies illustrate how crystallization within a lipid embedded environment allows novel insights, revealing the presence of bound cholesterol molecules that play important functional roles [21, 25]. These crystallographic methods are likely to continue to be critical in the determination of structures of the many important membrane proteins that are small and with little membrane peripheral mass.

## The bright lights of the femtosecond pulse

Recent years have also seen significant advances in the use of X-ray free electron laser (XFEL) methods. Unlike classical crystallography, in which single crystals are exposed to an X-ray beam multiple times while rotated, XFEL involves exposure of many thousands of randomly oriented microcrystals to femtosecond pulses of a high intensity X-ray beam, allowing a single diffraction image to be collected from each [27]. Complete datasets are then pieced together from images taken from thousands of crystals. This brings new challenges in data processing, which have been met by advances in software [28, 29, 30], but also brings the new opportunities that come from imaging tiny crystals without beam-induced radiation damage.

While early XFEL experiments involved structure-determination using molecular replacement, recent years have seen the extension of the method, allowing structures to be solved by experimental phasing. The first such proof-of-principle study used single-wavelength anomalous dispersion (SAD) phasing to re-solve the structure of the model system, lysozyme [31]. More recently, phasing by multiple isomorphous replacement with anomalous scattering (MIRAS) allowed determination of the structure of a novel protein, the BinAB larvicidal toxin using ∼300 nm long crystals purified directly from cells [32].

While advances in structure determination from tiny crystals are impressive, the real transformative potential of XFEL lies in the power that comes from imaging crystals at room temperature without beam-induced radiation damage. For the last twenty years, our model of classical crystallography has involved collection of multiple diffraction images from a single crystal that has been cryo-cooled to reduce damage from the X-ray beam. In contrast, XFEL can be conducted at room temperature, with one undamaged diffraction image obtained from each crystal. Comparison of structures of the serotonin transporter determined by XFEL or by classical crystallography shows that this can result in significant differences, with XFEL generating a structure presumably showing more authentic room-temperature dynamics [33]. Such damage-free XFEL measurements are also critical in understanding proteins with integral metal ions as X-ray induced photoreduction can affect the structures of metal centers and change the interpretation of their function. An example is the study of nitrite reductase by classical crystallography and by XFEL in which the damage-free XFEL structures show a different coordination structure around the metal ion and therefore support a different catalytic mechanism [34].

**Figure 3:**
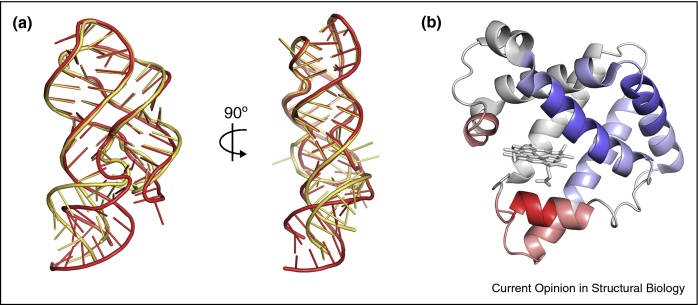
Conformational mobility studied using free electron lasers at room temperature. **(a)** Ligand binding induced changes in a riboswitch, rA71, studied by XFEL. The apo structure is shown in yellow, while the ligand-bound strucure is shown in red (PDB codes 5E54 and 5SWE) [38^•^]. **(b)** Time resolved XFEL investigation of structural changes in CO-bound myoglobin following ligand dissociation. Red indicates movement of the protein chain away from heme and blue movement toward heme. Adapted from [35^•^].

The ability to study tiny crystals at room temperature by XFEL also offers the opportunity to induce controlled changes, by the addition of ligands or by exposure to laser light, and to assess their effects on structure. For these experiments, small crystals can be better than large, as the time taken for a ligand to diffuse throughout the crystal will be minimized, and the potential stresses on the crystal lattice induced by conformational changes will be reduced. A recent example involves the photoactivation of crystals of myoglobin to break the Fe—CO bond [35^•^] (Figure 3a). The use of micrometer-sized crystals means that photons of light could reach the crystal center despite the absorption of some along the way, allowing all molecules to be activated simultaneously. The rapid collection of damage-free XFEL data shortly after activation therefore allowed structural characterization of the conformational changes that occur within 500 fs of CO release. Similar approaches have been used to study conformational transitions of photosystem II, allowing observation of the changes that occur during the formation of diatomic oxygen shortly after light activation [36] and to study the stages in the photocycle of fluorescent yellow protein [37]. The rapid diffusion of small molecule ligands into microcrystals has also allowed the development of ‘mix-and-inject’ XFEL, for example mixing a riboswitch with its ligand before XFEL, showing that such tiny crystals can occasionally undergo the most dramatic conformational transitions [38^•^] (Figure 3b).

Structure determination by XFEL is currently in its infancy with 57 structures deposited in the Protein Data Bank in 2016 (0.6% of that solved by classical crystallography in the same year). However, this number is likely to grow. Free electron lasers are in operation in California and at SPring-8 in Japan and the European X-ray laser is nearing completion. In addition, the ‘serial’ crystallography methods developed to fuel XFEL studies are now coming to more standard synchrotron beamlines [39, 40], allowing some of the same approaches to be performed at a wide range of weaker sources. These methods are unlikely to replace classical crystallography methods for high throughput structure determination any time soon. However, increased access to XFEL facilities will give researchers a new option for structure determination when they can only generate tiny microcrystals or nanocrystals, and will allow them to perform a range of intelligent experiments to characterize induced transitions of macromolecules as they move through their functional cycles.

## The role of crystallography in a time of revolution

Structural biology is in a time of flux with ‘the electron microscopy revolution’ exciting us all. Improvements in both the resolution obtained and reduction in the size of structures determined by electron microscopy have transformed the classical dogma of integrated structural biology. In the recent past, researchers aimed toward pseudo-atomic models of large complexes, in which they docked high-resolution crystal structures into moderate resolution single particle electron cryo-microscopy-derived maps. Now, for a number of systems, there is the potential to go straight to an atomic model by electron microscopy alone. Structure determination by electron microscopy also has the potential to avoid the extensive protein engineering that can be required to make a macromolecule crystallise, allowing structures to determined from microgram quantities of macromolecular complexes purified directly from natural sources, and for different conformational states to be ‘purified’ from each other computationally during structure determination [41, 42, 43, 44]. So where does crystallography stand and does it stand a chance?

In these early days of the revolution, crystallography plays a major role, with the vast majority of structures deposited in the Protein Data Bank still determined using this method. But the near future will see increased availability of electron microscopes and continued development of detectors and software, with likely further improvement in resolution and the determination of structures of decreasing size. So will crystallography continue to play a major part? In the years to come, the answer is clearly yes. If a macromolecular system is crystallizable, it is significantly quicker, easier, higher throughput and more accessible to solve its structure by crystallography. Even as electron microscopy continues to advance, we expect the importance of crystallography to continue, in particular due to fundamental differences in the mode of structure determination by the two methods. While crystallography relies on whether a molecule can be induced to assemble into an ordered array, electron microscopy relies on the ability of software to distinguish between different views of a macromolecular particle and to collect them together in classes to allow the averaging of signal from these noisy images [45]. For smaller, pseudo-symmetric particles, or those with a more spherical shape and where homologous structures are not available to bootstrap the alignment of raw images to a starting model, this will continue to be a significant challenge. Here, crystallography will continue to play a major role.

Crystallography will also continue to play a role due to the stabilizing effects of the crystal environment. Structures derived from electron microscopy often demonstrate variable resolution, with the ordered core of the molecule at sufficient resolution to build an atomic model, while flexibly linked peripheral regions are at lower resolution. This has clear advantages, in providing insight into the natural dynamics of a macromolecule, but can also preclude the determination of structures of small ordered domains flexibly attached to the core of a large complex. Crystallography will be able to define the structures of these regions, perhaps through their ordering within the crystal lattice. Alternatively this may be achieved by defining their boundaries by electron microscopy, followed by their crystallization and structure determination, and the docking of high-resolution structures back into the lower resolution regions of the electron microscopy reconstruction.

The mode of interaction of the imaging beam and the sample also provides a fundamental difference between electron microscopy and crystallography. While the electron beam of an electron microscope is deviated by the Coulomb potential of the constituent atoms, the photons of the X-ray beam interact with the electrons. The potential to tune the wavelength of the X-ray beam to match an electronic transition with a specific element therefore allows the application of anomalous scattering, allowing specific atoms to be identified within the structure. This, together with the exciting potential to study molecules at room temperature using XFEL, will open up numerous methods that can be used to study molecular transitions, which are not feasible by electron microscopy.

The future of structure biology is therefore bright, with transformative new technologies enabling a spectrum of exciting new discoveries, previously considered out of grasp. The tools and facilities available for X-ray crystallography are developing rapidly and the technique will continue to play an important role in future scientific discovery as part of an integrated structural biology approach, even as electron microscopy methods continue to advance. It is for this reason that the authors, while excitedly commissioning our new electron microscopes, are not decommissioning the crystallization robots.

## Conflict of interest

None declared.

## References and recommended reading

Papers of particular interest, published within the period of review, have been highlighted as:

• of special interest
